# Atlanta Medical College

**Published:** 1873-07

**Authors:** 


### ATLANTA MEDICAL COLLEGE.

A reorganization of the Faculty of this institution has been recently effected, which it is believed will add greatly to the prospects of placing it upon such a footing as to offer advantages to medical students which will bear a favorable comparison with most of the medical colleges of the country. Among the changes we note the appointment of Dr. G. W. Holmes, of Rome, Ga., to the chair of Principles and Practice of Medicine; Dr. Robert Battey, of the same city, to the chair of Obstetrics; Dr. A. W. Calhoun, (now and for the past two years in Europe,) to the chair of Diseases of the Eye and Ear. The senior editor of this JOURNAL has also been elected to the chair of Clinical Medicine; and in this connection it is perhaps proper to say that having retired from medical teaching for a number of years with the purpose of not being again connected with it, he has only been induced to change his determination by the urgent solicitation of those who believe that they see a most favorable prospect for the establishment of an institution which will not only be a credit to the city and the State, but will aid in placing medical education upon the highest possible basis. The perfect harmony prevailing throughout the entire profession of the State, and the pleasant relations existing between the several medical institutions—all manifesting a common purpose to *unite* in advancing the true interests of the profession—has enabled him to take a more hopeful view of the prospect and to reëngage with interest in a work which he had supposed was abandoned forever.

